# T Cell Post-Transcriptional miRNA-mRNA Interaction Networks Identify Targets Associated with Susceptibility/Resistance to Collagen-induced Arthritis

**DOI:** 10.1371/journal.pone.0054803

**Published:** 2013-01-24

**Authors:** Paula B. Donate, Thais A. Fornari, Claudia Macedo, Thiago M. Cunha, Daniele C. B. Nascimento, Elza T. Sakamoto-Hojo, Eduardo A. Donadi, Fernando Q. Cunha, Geraldo A. Passos

**Affiliations:** 1 Molecular Immunogenetics Group, Department of Genetics, Faculty of Medicine of Ribeirão Preto, University of São Paulo, Ribeirão Preto, Brazil; 2 Inflammation and Pain Group, Department of Pharmacology, Faculty of Medicine of Ribeirão Preto, Ribeirão Preto, Brazil; 3 Department of Biology, Faculty of Philosophy, Sciences and Letters of Ribeirão Preto, Ribeirão Preto, Brazil; 4 Division of Clinical Immunology, Department of Medicine, Faculty of Medicine of Ribeirão Preto, Ribeirão Preto, Brazil; 5 Disciplines of Genetics and Molecular Biology, Department of Morphology, Faculty of Dentistry of Ribeirão Preto, Ribeirão Preto, Brazil; University of British Columbia, Canada

## Abstract

**Background:**

Due to recent studies indicating that the deregulation of microRNAs (miRNAs) in T cells contributes to increased severity of rheumatoid arthritis, we hypothesized that deregulated miRNAs may interact with key mRNA targets controlling the function or differentiation of these cells in this disease.

**Methodology/Principal Findings:**

To test our hypothesis, we used microarrays to survey, for the first time, the expression of all known mouse miRNAs in parallel with genome-wide mRNAs in thymocytes and naïve and activated peripheral CD3^+^ T cells from two mouse strains the DBA-1/J strain (MHC-H2q), which is susceptible to collagen induced arthritis (CIA), and the DBA-2/J strain (MHC-H2d), which is resistant. Hierarchical clustering of data showed the several T cell miRNAs and mRNAs differentially expressed between the mouse strains in different stages of immunization with collagen. Bayesian statistics using the GenMir^++^ algorithm allowed reconstruction of post-transcriptional miRNA-mRNA interaction networks for target prediction. We revealed the participation of **miR-500**, **miR-202-3p** and miR-30b*, which established interactions with at least one of the following mRNAs: Rorc, Fas, Fasl, Il-10 and Foxo3. Among the interactions that were validated by calculating the minimal free-energy of base pairing between the miRNA and the 3′UTR of the mRNA target and luciferase assay, we highlight the interaction of miR-30b*-Rorc mRNA because the mRNA encodes a protein implicated in pro-inflammatory Th17 cell differentiation (Rorγt). FACS analysis revealed that Rorγt protein levels and Th17 cell counts were comparatively reduced in the DBA-2/J strain.

**Conclusions/Significance:**

This result showed that the miRNAs and mRNAs identified in this study represent new candidates regulating T cell function and controlling susceptibility and resistance to CIA.

## Introduction

Rheumatoid arthritis (RA) is a systemic autoimmune disorder characterized by inflammation of the synovial tissue that can lead to destruction of bone and cartilage, eventually leading to disability [1], [2]. The mechanisms involved in disease initiation and progression are still incompletely understood, as RA has a complex component controlled by several genes that interact together with environmental and stochastic factors [2].

A hallmark feature of RA pathology is the infiltration and accumulation of T cells in the synovial tissue [3]. The T cells isolated from the synovial and joint tissue show an activated and memory phenotype. These cells appear to respond poorly to stimulation with mitogen or antigens in vitro and fail to undergo apoptosis [4]. The mechanisms underlying the impaired apoptosis of T cells in RA remain largely unclear. Inhibition of apoptosis causes these cells to accumulate in both the synovia and the periphery. Due to the inherent difficulty in studying these questions in humans, researchers use collagen-induced arthritis (CIA) in mice as an animal model of autoimmune inflammatory polyarthritis reproducing many of the clinical and pathological features of human RA [5]. Similar to RA, susceptibility to CIA has a genetic basis that is associated with certain MHC-II alleles (H2-Aq and H2-Ar) that make certain mouse strains susceptible [6].

The molecular genetics of RA and CIA is an emerging field with contributions from our group [7]–[10] and from others [11], [12] that have demonstrated an association between transcriptional expression profiles of mRNAs and RA. Recent data suggest that certain microRNAs (miRNAs) in T cells might play a role in the onset of this disease [13], and several groups have focused their attention on the role played by miRNAs in the pathogenesis of RA, as well as their potential use as biomarkers to monitor the disease [14], [15].

MiRNAs have emerged as post-transcriptional regulators of gene expression in a variety of biological processes [16]–[19]. They regulate protein expression via complementary sequence recognition of the 3′ untranslated region (3′UTR) of the target mRNA, triggering the degradation of the target transcript if miRNA-mRNA hybridization is faultless [20]–[22]. A recent paper indicated that the major effect of miRNAs is to decrease mRNA levels [23]. MiRNAs can potentially regulate hundreds of proteins [24] and modulate the concentration of proteins over a narrow range in a dose-dependent manner [25].

These molecules are involved in hematopoietic cell function and development, and a few miRNAs have been linked to specific T lymphocyte mechanisms. For example, miR-181a [26], miR-181c [27], miR-155 [28], miR-150 [29], miR-146 [13], and miR-142 [30] regulate T cell sensitivity to antigen stimulation, transcription factors, and activation-induced cell death.

Different miRNA expression patterns in RA patients and healthy controls or patients affected by osteoarthritis [14], [31] have been the focus of many studies. Most studies have examined miRNA expression in plasma and serum, while others focused mainly on tissue analysis [15]. Several miRNAs were identified in the T cells of RA patients. MiR-223 is upregulated in CD4^+^ naïve T lymphocytes [32]. As T lymphocytes are considered to play a role in the pathogenesis of RA, these results suggest that this miRNA could influence the T cell response and therefore contribute to disease onset. A recent study showed that miR-363 and miR-498 are downregulated in the CD4^+^ T cells of RA patients, while miR-146a expression is significantly upregulated [13].

In this study, we used a multidimensional approach to integrate genome-wide miRNA and mRNA expression. We surveyed, for the first time, the expression of mouse miRNA sequences in thymocytes, naïve and activated T cells in two mouse strains, the DBA-1/J strain, which is susceptible to CIA, and the DBA-2/J refractory strain. In parallel, we profiled global mRNA levels, including the entire mouse genome (transcriptome profiling). Using the GenMiR^++^ program, which is a Bayesian statistics-based data analysis algorithm and designed to reconstruct miRNA-mRNA networks interactions, important mRNAs related to the immune system, T cell function and differentiation were found as potential targets.

The majority of miRNA-mRNA interactions were validated by calculations of the minimal Gibbs free-energy using the RNA-Hybrid algorithm [33], [34] and two of them (miR30b*-Rorγt mRNA and miR196b-CD8a mRNA) were confirmed to occurs within the cellular milieu by the luciferase assay. We highlighted miR-30b* interacting with RORγt mRNA because the encoded protein (RORγt) plays a role as a transcription factor involved in Th17 cell differentiation [35]. We observed that both RORγt mRNA and protein were down-regulated in the DBA-2/J strain, which is refractory to CIA. Moreover, the proportion of Th17 cells in this strain is lower than that found in the DBA-1/J strain.

In this study we identified novel miRNAs involved in T cell function in the context of autoimmunity of CIA model-system and provided evidence for their role in post-transcriptional networks involving key mRNAs. Evidence was taken that modulation of which might control the susceptibility/resistance to CIA in mice.

## Materials and Methods

### Animals

Male DBA/1J and DBA-2/J mice (4 and 12 weeks old) weighing 18–22 g each were housed in temperature-controlled rooms (22°C) in the animal facility of the School of Medicine of Ribeirão Preto, University of São Paulo, Ribeirão Preto, Brazil, and received water and food *ad libitum*. All experimental protocols were approved by the local Ethical Committee on Animal Experimentation of the Faculty of Medicine of Ribeirão Preto (Permit Number: 119/2008).

### Induction and Assessment of Collagen-induced Arthritis

Male DBA/1J and DBA-2/J mice received 200 µg of a chicken type II collagen (Sigma-Aldrich St. Louis, MO, USA) emulsion in complete Freund’s adjuvant (CFA) by intradermal injection (day 0). A second injection of collagen (200 µg dissolved in sterile apyrogenic pH 7.2 phosphate buffered saline, (PBS) was given on day 21 by intradermal injection [36]. Mice were monitored daily for signs of arthritis for which severity scores were derived as follows: 0 = normal paw, 1 = paw with erythema, 2 = paw with erythema plus swelling, and 3 = paw with extension/loss function. The total score indicated the sum of the individual scores for all four limbs. Disease onset characterized by erythema and/or paw swelling was observed between days 25 and 35. The control group corresponded to the sham-immunized mice, which only received an injection of CFA.

### Thymocytes and CD3^+^ T cell Isolation

The thymi from five 4-week-old male DBA-1/J and DBA-2/J mice were dissected and trimmed of fat and connective tissue in DMEM/F10 medium, and thymocytes were obtained by 2–3 passages of the thymic fragments throughout a 10-*µ*m mesh nylon membrane (Sefar Inc. Depew, NY, USA). Pelleted thymocytes were suspended in PBS. Fluorescent-activated cell sorting (FACS) analysis in a BD-FACScalibur flow cytometer with a phycoerythrin (PE)-labeled anti-CD3 antibody indicated that this procedure yielded approximately 93% purity of the thymocyte population. These cells were then used for total RNA preparation. The peripheral CD3^+^ T lymphocytes from five 4-week-old non-immunized mice (here, considered as naïve) and five 12-week-old control and collagen immunized male mice of both strains were isolated from the inguinal lymph nodes and spleens using magnetic beads for negative selection (Pan T-cell isolation kit, mouse, Miltenyi Biotec), according to the manufacturer’s instructions. The FACS analysis with PE-labeled anti-CD3 antibody indicated that this procedure yielded a CD3^+^ T lymphocyte population of approximately 88% purity. These cells were then used for total RNA preparation. All hybridizations of RNA pools were performed in triplicate for mRNA or in duplicate for miRNA microarrays.

### Total RNA Extraction

The total RNA was extracted from approximately 1×10^7^ thymocytes or peripheral T CD3+ lymphocytes using the mirVana total RNA isolation kit (Ambion, NY, USA), according to the manufacturer’s instructions. RNA preparations were confirmed to be free of proteins and phenol by UV spectrophotometry. RNA degradation was assessed by microfluidic electrophoresis using Agilent 6000 RNA Nano chips and an Agilent 2100 Bioanalyzer (Agilent Technologies, Santa Clara, CA, USA). Only RNA samples that were free of proteins and phenol and featured an RNA Integrity Number (RIN) ≥9.0 were used.

### Microarray Hybridizations

#### mRNA expression profiling using agilent 4×44 K mouse oligo arrays

Individual 500 ng total RNA were used to synthesize double-stranded cDNA and cyanine 3 (Cy3)-CTP-labeled complementary amplified RNA (cRNA) using the Agilent Linear Amplification Kit (Agilent Technologies, Santa Clara, CA, USA), according to the manufacturer’s instructions. Cyanine-labeled complementary RNA was hybridized to microarrays in SureHyb chambers (Agilent) in a rotator oven using Agilent mouse 4×44 K oligonucleotide microarrays (Agilent Technologies) for 18 h at 60°C. Each array contained 44,000 oligonucleotide probes covering the entire mouse functional genome plus internal control probes. The arrays were washed according to the manufacturer’s instructions and scanned using an Agilent DNA Microarray scanner.

### miRNA Expression Profiling using Agilent 8×15 K Mouse Oligo Arrays

The total RNA samples were Cy-3-labeled using the Agilent miRNA Complete Labeling and Hybridization Kit (Agilent Tech, Mississauga, ON, Canada). Briefly, 100 ng of total RNA was dephosphorylated by incubation with calf intestinal phosphatase at 37°C for 30 min, denatured in 100% DMSO at 100°C for 5 minutes and labeled with perCp-Cy3 using T4 ligase at 16°C for 1 h. Each labeled RNA sample was hybridized to an individual array on an 8×15 K format Agilent mouse miRNA array slides. Each array contained probes for 567 mouse miRNAs and 10 mouse gamma herpes virus miRNAs. Hybridizations were performed in SureHyb chambers (Agilent) for 20 h at 55°C, washed according to the manufacturer’s instructions and scanned.

### Microarray Data Analysis

The oligo-mRNA and oligo-miRNA array slides were scanned using a DNA microarray scanner (Agilent Technologies), and the hybridization signals were extracted using the Agilent Feature Extraction software version 10.5. Gene expression profiles from independent preparations of thymocytes (from 4-week-old DBA-1/J and DBA-2/J mice) or CD3^+^ peripheral lymphocytes (from 4 and 12-week-old DBA-1/J and DBA-2/J mice) were analyzed by comparing the microarray hybridizations of the respective samples. Figure S1 depicts the experimental design for further comparison of the gene profiling. The microarray numerical quantitative data were normalized to the 75th percentile and were analyzed using the GeneSpring GX bioinformatics platform (http://www.agilent.com/chem/genespring) according to default instructions allowing for hierarchical clustering of samples of mice or genes based on an ANOVA statistical analysis (*P<0.05*), fold change ≥2.0 and uncentered Pearson correlation metrics [37].

The similarities and dissimilarities in gene expression (mRNA and miRNA) are presented as dendrograms in which the pattern and length of the branches reflect the relatedness of the samples or genes and heat maps. Gene ontology annotations were retrieved from the GeneSpring platform and were used to assess the biological functions of mRNAs based on a corrected p value <0.05 and cut-off = 0.1. Information about the miRNAs was retrieved from the miRBase data bank (www.mirbase.org).

A complete file that provides all of the mRNAs and miRNAs present in the arrays used in this study, as well as the experimental conditions, is available online at the MIAME public database (http://www.ebi.ac.uk/miamexpress), Array Express accession E-MEXP-3369 (for mRNA hybridizations) and E-MEXP-3370 (for miRNA hybridizations).

### Reconstruction of miRNA-mRNA Post-transcriptional Interaction Networks

We used the GenMiR^++^ algorithm to reconstruct the miRNA-mRNA interaction networks, which use as a basis the respective miRNA and mRNA expression profiles from the same sets of tissues or cell types to identify candidate miRNA-mRNA target pairs that are best supported by the expression data, and a database of a potential set of mRNA targets for each miRNA identified [38]. In this study, we used the TargetScanS database (http://www.targetscan.org/) to select the target mRNA set.

For each mRNA differentially expressed, the candidate miRNA regulators were scored according to how much the miRNA expression profile contributed to explaining the down-regulation of the mRNA expression, given all other miRNA candidates.

GenMiR^++^ calculates the scores by attempting to reproduce an mRNA’s profile by a weighted combination of the genome-wide average normalized expression profile and the negatively weighted profiles of a subset of the miRNA regulators. The miRNAs that often appear in subsets whose reproductions are good fits to the mRNA profile are assigned the highest scores. The scores are plotted and a reasonable threshold was determined to select no more than the top 20% of interactions predicted by GenMiR++. The graphical Cytoscape v 2.1 program (www.cytoscape.org) was used to design the networks.

### Validations of miRNA-mRNA Interactions

#### Determination of hybridization minimum free energy

Observing the miRNA-mRNA interaction networks generated by the GenMiR^++^ algorithm, we selected pairs based on the biological function of the mRNA target. The RNA-hybrid algorithm [33], [34], which is available at (http://bibiserv.techfak.uni-bielefeld.de/rnahybrid), was then used to validate the annealing. This method involves a dynamic programming algorithm that calculates the most favorable hybridization between a given miRNA and its mRNA target, calculating the minimum free energy (MFE) based on a thermodynamic state that postulates that an RNA duplex is more stable and thermodynamically stronger when the free energy is low [39].

#### Luciferase reporter assay

The oligonucleotide pairs containing a portion of the Rorc or CD8a 3′UTR predicted binding site for miRNAs were synthetized by Sigma-Aldrich, St. Louis, MO, USA. The oligonucleotides were annealed and cloned into the pmirGLO Vector (Promega Corporation, USA) between the XhoI/XbaI restriction sites resulting in the miRNA target region in the correct 5′ to 3′ orientation, immediately 3′ downstream of the luciferase gene. For selected targets, we introduced point mutations into the 7-nt seed-binding sequence. These constructs, named “pMIR-Rorc” or “pMIR-CD8a” for the wild-type sequences and “pMIR-Rorc(m)” or “pMIR-CD8a(m)” for the mutant mismatch sequences were selected by colony-polymerase chain reaction (PCR) using the following pair of primers: forward 5′AGGTTACAACCGCCAAGAAG3′ and reverse 5′CAGCCAACTCAGCTTCCTTT 3′, which flank the vector polycloning site.

For the luciferase reporter assay, 0.2 ug of each pmirGLO constructs was transfected together with 1.6 pmol of miR-30b*, miR-196b or scrambled miRNA (Thermo Scientific Dharmacon, Waltham, MA, USA) into HEK-293T cells (6×10^4^ cells/well) in a 96-well plate. Transfections were done using Attractene Transfection Reagent (Qiagen, Hilden, Germany) according to the manufacturer’s instructions. Transfected cells were incubated at 37°C in a 5% CO_2_ incubator and 24 hours after transfections, cells were lysed in Passive Lysis Buffer and then firefly and renilla luciferase activities were measured in a Molecular Devices FlexStation 3 Luminometer with the Dual-Luciferase reporter system (Promega Corporation, USA) according to the manufacturer’s instructions. Our laboratory has a CTNBio biosafety permission (Permit No. 0040/98).

### Flow Cytometry

Spleen and lymph nodes were prepared as single-cell suspensions according to standard protocols, and the following monoclonal antibodies (BD Biosciences, New Jersey, USA) were used for staining: CD4 PerCP and CD8 FITC. For intracellular detection of RORγt, cells were first treated with anti-CD4 and then fixed, permeabilized, and stained with monoclonal RORγt PE. Flow cytometry was performed in a FACSCalibur instrument (Becton Dickinson, New Jersey, USA) and analyzed with FlowJo software (TreeStar, Ashland, USA).

### Statistical Analysis

The real-time PCR and FACS results are presented as standard error of the mean (SEM). The differences were evaluated by one-way ANOVA followed by Student’s t-test (two groups) or Bonferroni’s test (three or more groups), *P*<0.05, *P*<0.01, and *P*<0.001 were considered statistically significant.

## Results

### Collagen-induced Arthritis (CIA)

Immunization with type II collagen induced CIA in male DBA-1/J mice, whose CIA scores reached a peak on day 35. Male DBA-2/J mice injected with the same schedule did not exhibit any features of arthritis development. Male mice from both strains immunized with only CFA were used as controls (data not shown).

### miRNA Expression Profile in Thymocytes and Peripheral T Cells

**Figure 1:**
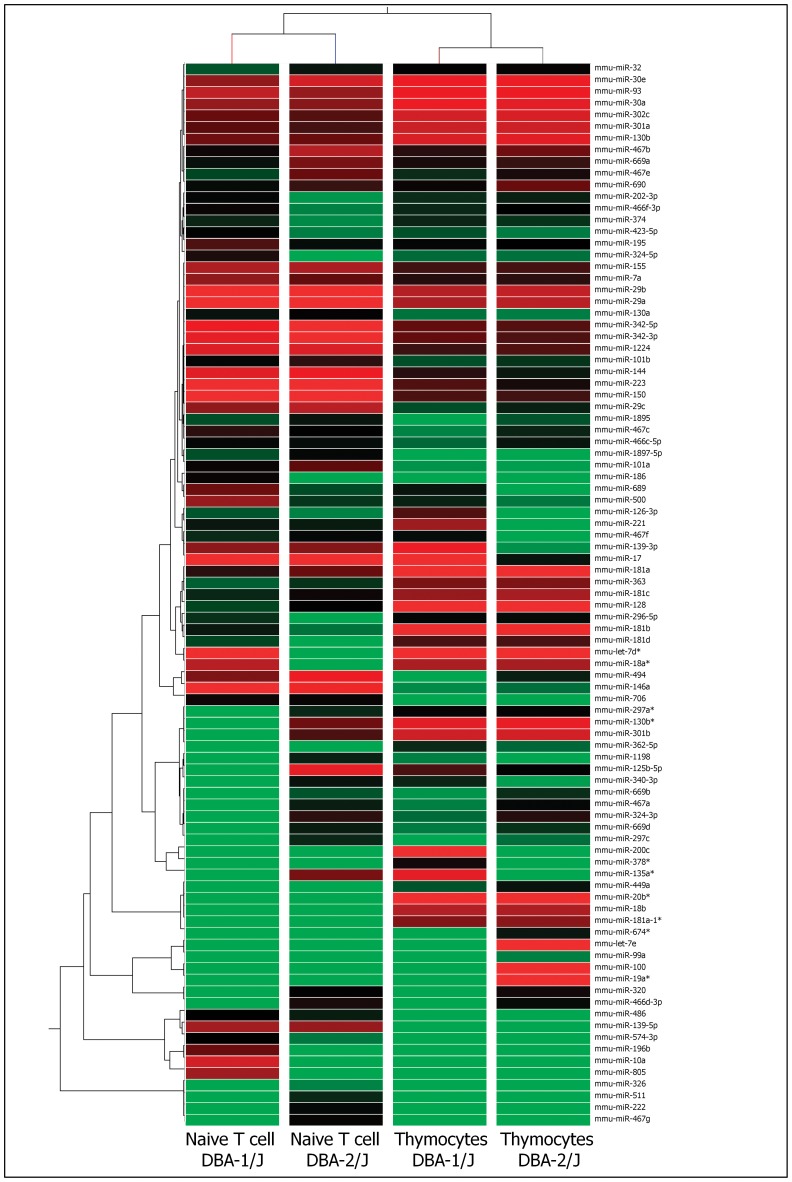
Modulation of miRNAs as evaluated by microarrays. Hierarchical clustering and color heat-map of miRNome (miRNAs) of thymocytes isolated from 4-week-old DBA-1/J and DBA-2/J mice and CD3^+^ T cells from the spleen and lymph nodes of 4-week-old mice from both strains based on microarray expression profiling. The dendrogram and heat-map were obtained using cluster and tree-view algorithms through Agilent GeneSpring platform. Heat map legend: Red = up regulation, green = down regulation, black = unmodulated (Pearson correlation metrics).

We first compared miRNA expression in thymocytes and peripheral naïve CD3^+^ total T cells from 4-week-old DBA-1/J and DBA-2/J non-immunized animals (Figure 1). We next compared miRNA expression in activated CD3^+^ T cells from the spleen and lymph nodes of 12-week-old mice that were immunized with collagen.

**Figure 2:**
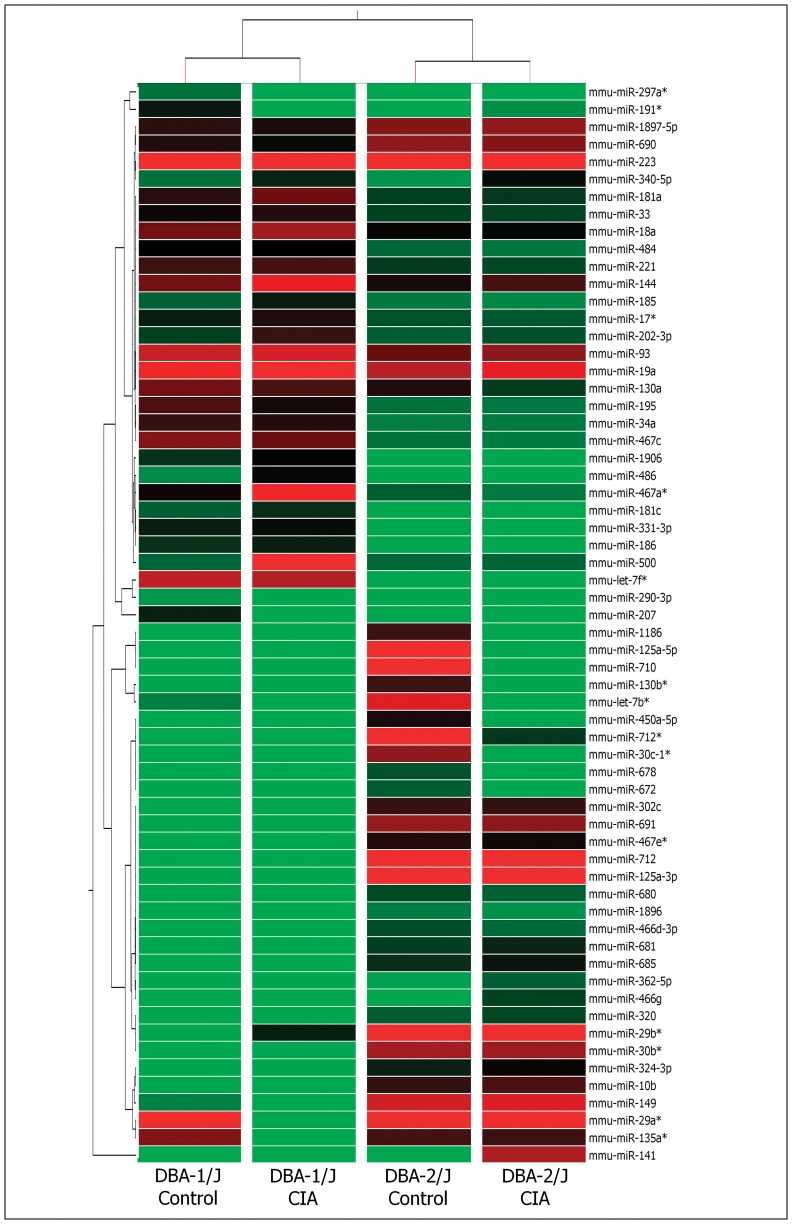
Modulation of miRNAs as evaluated by microarrays during collagen induced arthritis (CIA). Hierarchical clustering and color heat-map of miRNome (miRNAs) of CD3^+^ T cells from the spleen and lymph nodes of 12-week-old mice from the resistant DBA-2/J and the susceptible DBA-1/J strain, both immunized with chicken type II collagen, based on microarray expression profiling. The control mice were immunized just with complete Freund’s adjuvant (CFA) without any collagen. The dendrogram and heat-map were obtained using cluster and tree-view algorithms through Agilent GeneSpring platform. Heat map legend: Red = up regulation, green = down regulation, black = unmodulated (Pearson correlation metrics).

We identified 91 differentially expressed miRNAs (p<0.05, fold change ≥2.0) comparing thymocytes to naïve peripheral CD3^+^ T cells from the DBA-1/J and DBA-2/J mouse strains (Figure 1), and 62 differentially expressed miRNAs (p<0.05, and fold change ≥2.0) comparing activated CD3^+^ T cells during CIA development (Figure 2). We selected the specific up-regulated miRNAs from each group for subsequent of miRNA-mRNA interaction prediction. Three miRNAs (miR-126-3p, miR-221 and miR-200c) were exclusively up-regulated in thymocytes of DBA-1/J strain, and three others (miR-let-7e, miR-100 and miR-19a*) were exclusively up-regulated in the DBA-2/J strain.

In naïve peripheral CD3^+^ T cells, we found six miRNAs exclusively up-regulated in DBA-1/J mice (miR-195, miR-689, miR-500, miR-196b, miR-10a and miR-805) and four exclusively up-regulated in DBA-2/J mice (miR-467e, miR-101a, miR-125-5p and miR-669a).

During the course of CIA, six miRNAs (miR-181a, miR-144, miR-17*, miR-202-3p, miR-467a* and miR-500) were up-regulated in peripheral CD3^+^ T lymphocytes of DBA-1/J strain. In the peripheral CD3^+^ T lymphocytes of DBA-2/J strain, we found 11 miRNAs (miR-302c, miR-691, miR-712, miR-125a-3p, miR-29b*, miR-30b*, miR-10b, miR-149, miR-141, miR-1897-5p and miR-690) that were up-regulated.

### Messenger RNAs Differentially Expressed in Thymocytes and Peripheral T cells

Although the expression pattern remained unchanged during CIA development for the majority of the 44,000 sequences tested, 14,230 were differentially expressed in thymocytes compared to naïve T cells, and 3,155 were differentially expressed compared to activated CD3^+^ T cells.

**Figure 3:**
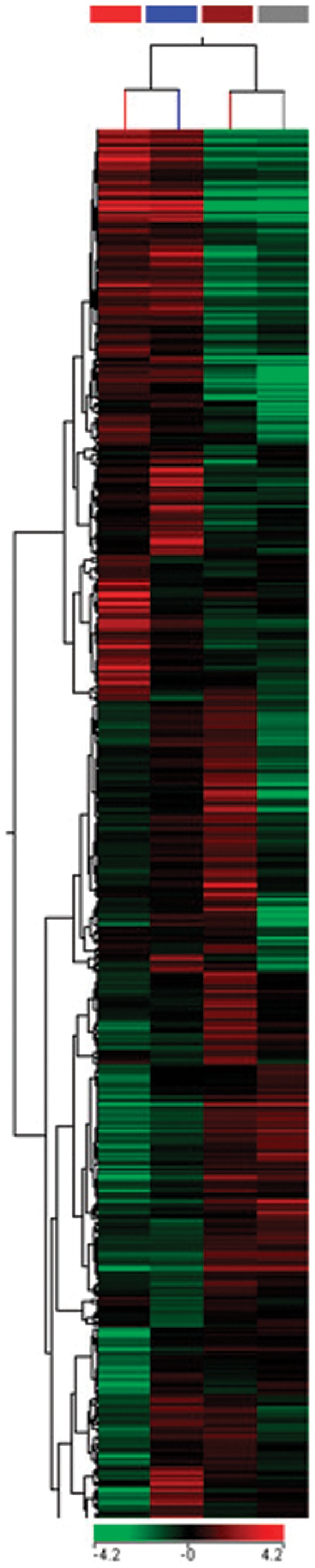
Modulation of mRNAs as evaluated by microarrays. Hierarchical clustering and color heat-map of the transcriptome (mRNAs) of thymocytes isolated from 28-day-old DBA-1/J and DBA-2/J mice and CD3^+^ T cells from the spleen and lymph nodes of 4-week-old mice from both strains based on microarray expression profiling. The dendrogram and heat-map were obtained using cluster and tree-view algorithms through Agilent GeneSpring platform. This figure shows the modulation of genes related to immune system based on Gene Ontology (GO terms). Heat map legend: Red = up regulation, green = down regulation, black = unmodulated (Pearson correlation metrics). Sample dendrogram legend: red-line = Naïve T cell DBA-1/J; blue-line = Naïve T cell DBA-2/J; brown-line = Thymocytes DBA-1/J; gray-line = Thymocytes DBA-2/J.

**Figure 4:**
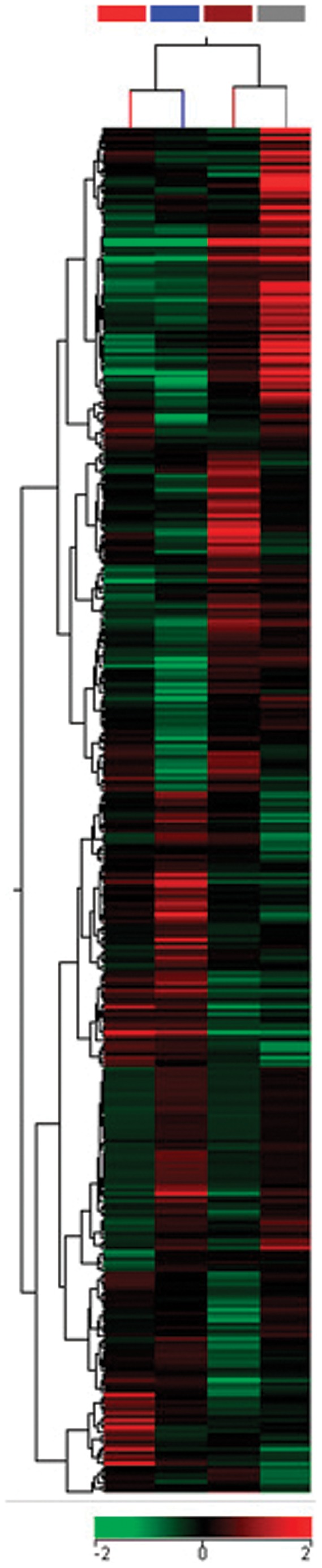
Modulation of mRNAs as evaluated by microarrays during collagen induced arthritis (CIA). Hierarchical clustering and color heat-map of the transcriptome (mRNAs) of CD3+ T cells from the spleen and lymph nodes of 12-week-old mice from the resistant DBA-2/J strain and the susceptible DBA-1/J strain immunized with chicken type II collagen, based on microarray expression profiling. The control mice were immunized just with complete Freund’s adjuvant (CFA) without any collagen. The dendrogram and heat-map were obtained using cluster and tree-view algorithms through Agilent GeneSpring platform. This figure shows the expression of genes related to immune system function based on Gene Ontology (GO terms). Heat map legend: Red = up regulation, green = down regulation, black = unmodulated (Pearson correlation metrics). Sample dendrogram legend: red-line = DBA-1/J control; blue-line = DBA-1/J CIA; brown-line = DBA-2/J control; gray-line = DBA-2/J CIA.

Functional annotation of the differentially expressed mRNAs using Gene Ontology (GO) (http://www.geneontology.org/) identified 1,813 immune system-related mRNAs among the 14,230 significantly expressed in thymocytes compared to naïve T cells (Figure 3) and 486 immune system-related mRNAs in CD3^+^ T cells during CIA (Figure 4). Hierarchical clustering revealed that these sequences had distinct expression patterns. These sequences were then used to reconstruct miRNA-mRNA interaction networks to identify potential targets.

### Reconstruction of miRNA-mRNA Interaction Networks

The miRNA-mRNA interaction networks predicting targeted immune system mRNAs in thymocytes and peripheral naïve and activated CD3^+^ T cells from the DBA-1/J and DBA-2/J mouse strains were depicted and the selected miRNAs and their targets are shown in Table 1.

**Table 1:** Up-regulated microRNAs in thymocytes and CD3+T lymphocytes in DBA-1/J and DBA-2/J mice strains and their potential targets related to immune system predicted by GenMiR++.

MicroRNAs	Potencial targets
miR-221	Add2, Ank1, Camp, Meis1, Pawr, S100a9, Sox6, Tlr3
miR-126-3p	Add2, Ank1, Camp, Epb4.2, Meis1, Pawr, Sox6, Spna1, Tlr3
miR-200c	Add2, Ahsp, Ank1, Bcl11a, Car2, Cd55, Cd59a, Cd59b, Cd83, Ctsg, Cxcl9, Enpp3, Epb4.2, Epor, Hspa1b, Igj, Il1rl1, Klf1, Lmo2, Meis1, Mpo, Ncf1, Pawr, Ptger3, Snca, Sox6, Sphk1, Spna1, Spon2, Tal1, Tlr3, Trim10, Zbtb32
miR-let-7e	Add2, Adora2b, Ahsp, Ank1, C4bp, Cd59a, Csf1r,Defb6, Enpp3, Epas1, Epb4.2, Fyb, Ghr, Gm5077, Gnas, Grin2b, H28, H2-Q10, Hoxa9, Klf1, Lifr, Lmo2, Mecom, Meis1, Mpl, Ms4a2, Pawr, Pbx1, Snca, Sox6, Sphk1, Spna1, Tal1, Tgfb2, Tlr3, Trim10
miR-100	Add2, Adora2b, Ahsp, Ank1, Cd59a, Csf1r, Ctsg, Defb6, Enpp3, Epas1, Epb4.2, Epor, Fyb, Ghr, Gm5077, Grin2b, H28, H2-Q10, Hdac9, Hoxa9, Il1rl1, Klf1, Lifr, Lmo2, Meis1, Mpl, Mpo, Ms4a2, Pawr, Smap1, Snca, Sox6, Sphk1,Spna1,Tal1, Tgfb2, Tlr3, Trim10, Zbtb7a
miR-19a*	Add2, Ahsp, Ank1, Cd59a, Csf1r, Defb6, Enpp3, Epas1, Epb4.2, Epor, Fyb, Gm5077, Grin2b, H28, H2-Q10, Hdac9, Hoxa9, Klf1, Lifr, Lmo2, Meis1,Mpl, Ms4a2,Pawr, Snca, Sox6, Sphk1, Spna1, Tal1, Tgfb2, Tlr3, Trim10
miR-195	Add2, Ank1, Camp, Enpp3, Epb4.2, Meis1, Ms4a2, Pawr, S100a9, Sox6, Spna1, Tlr3
miR-689	Add2, Ank1, Camp, Endou, Ly6d, Meis1, Pawr, Rag1, S100a9, Sox6, Tlr3
miR-500	Add2, Ank1, Camp, Meis1, Pawr, Rag1, S100a9, Sox6, Tlr3
miR-196b	Bcl11b, Camp, Ccl12, Cd247, Cd8a, Endou, Il5ra, Lig4, Ly6d, Mr1, Pax1, Rag1, Rag2, Rorc, S100a9, Sh2d1a, Themis, Thy1
miR-10a	Bcl11b, C1qc, Camp, Ccl12, Ccl24, Ccl8, Cd247, Cd4, Cd8a, Cfb,Endou, Esr2, Il5ra, Lig4, Ly6d, Mr1, P2rx1, Pax1, Rag1, Rag2, Rorc, Rtkn2, S100a9, Satb1, Sh2d1a, Sla2, Socs1, Sox4, Themis, Thy1
miR-805	Bcl11b, C1qc, Camp, Ccl12, Ccl24, Cd247, Cd8a, Endou, Esr2, Il5ra, Lifr, Lig4,Ly6d, Rag1, Rag2, Rorc, S100a9, Themis, Thy1
miR-125-5p	Add2, Adora2b, Ank1, Cd59a, Enpp3, Epas1, Epb4.2, H28, Hspa1b, Klf1, Lmo2, Meis1, Ms4a2, Pawr, Pbx1, Sox6, Sphk1, Spna1, Tlr3
miR-467e	Add2, Ank1, Camp, Enpp3, Epb4.2, H28, Meis1, Ms4a2, Pawr, Sox6, Spna1, Tlr3
miR-669a	Add2, Adora2b, Ank1, Camp, Enpp3, Epas1, Epb4.2, Meis1, Ms4a2, Pawr, Pbx1, S100a9, Sox6, Spna1, Tlr3
miR-101a	Add2, Ank1, Camp, Endou, Ly6d, Meis1,Pawr, Rag1, Rag2, S100a9, Sox6
miR-144	Aicda, Azgp1, Bank1, Btla, Cbfb, Cd1d2, Cd247, Cd38, Cd79b, Cx3cr1, Cxcr4, Cxcr5, Fcgr2b, Gimap5, Gnas, H2-Ab1, H2-DMb2, H2-Ea, H2-Eb1, H2-T3, Hdac9, Hspa1b, Icam1, Igh-6, Il1rap, Inhba, Klhl6, Ly6d, Lyn, Mll5, Ms4a1, Mx1, Mx2, Oas1b, Orm2, Pdcd1lg2, Pou2f2, Prkca, Prkcd, Ptprc, Ripk2, Rogdi, Sh2d1b1, Spib, Tlr1, Tlr7, Tnfrsf13c, Twsg1, Fas, Gzma, Gzmm, Il10
miR-17*	Bad, Btla, Cd28, Cd38, Csf1r, Cxcr4, Dock2, Gnas, Hdac9, Hspa1b, Kdr, Klhl6, Lyn, Mll5, Nfkb1, Orm2, Pdcd1lg2, Pecam1, Pou2f2, Ptprc, Rab27a, Rogdi, Rsad2, Sh2b3, Sh2d1b1, Slc11a1, Tlr1, Tlr2, Tlr7
miR-181a	Aicda, Bank1, Btla, Cbfb, Cd79b, Cx3cr1, Gnas, H2-Ab1, H2-DMb2, H2-Ea, H2-Eb1, H2-T3, Hdac9, Hspa1b, Icam1, Il1rap, Inhba, Klhl6, Lyn, Ms4a1, Mx1, Orm2, Pdcd1lg2, Prkcd, Ptprc, Ripk2, Sh2d1b1, Spib, Tnfrsf13c, Gzma, Gzmm, Il10
miR-202-3p	Aicda, Azgp1, Bank1, Btla, C3, Cbfb, Cd1d2, Cd247, Cd38, Cd79b, Cx3cr1, Cxcr4, Cxcr5, Ddx58, Fcgr2b, Gimap5, Gnas, H2-Aa, H2-Ab1, H2-DMa, H2-DMb2, H2-Ea, H2-Eb1, H2-T3, Hdac9, Hspa1b, Hspa1b, Icam1, Igh-6, Il1rap, Il2ra, Inhba, Klhl6, Ly6d, Lyn, Mll5, Ms4a1, Mx1, Mx2, Oas1b, Orm2, Pbx1, Pdcd1lg2, Pou2f2, Prkca, Prkcd, Ptprc, Ripk2, Rogdi, Sh2d1b1, Spib, Tlr1, Tlr7, Tnfrsf13c, Twsg1, Fas, Gzma, Gzmm, Il10
MicroRNAs	Potencial targets
miR-467a*	Aicda, Azgp1, Bad, Bank1, Blnk, Btla, C3, Cbfb, Cd1d2, Cd247, Cd28, Cd38, Cd55, Cd79b, Clcf1, Csf1r, Cx3cr1, Cxcr4, Cxcr5, Ddx58, Dock2, Fcgr2b, Gimap5, Gnas, H2-Aa, H2-Ab1, H2-DMa, H2-DMb1, H2-DMb2, H2-Ea, H2-Eb1, H2-T3, Hdac9, Hoxb3, Hspa1b, Icam1, Igh-6, Il1rap, Il2ra, Inhba, Kdr, Klhl6, Lat2, Ly6d, Lyn, Mll5, Ms4a1, Mx1, Mx2, Myo1e, Nfam1, Nfkb1, Oas1b, Orm2, Pbx1, Pdcd1lg2, Plcg2, Pou2f2, Prkca, Prkcd, Ptprc, Ripk2, Rogdi, Rsad2, Selp, Sfpi1, Sh2b3, Sh2d1b1, Slc11a1, Spib, Tapbp, Tlr1, Tlr7, Tnfrsf13c, Twsg1, Ywhaz, Fas, Fasl, Gzma, Gzmm
miR-500	Aicda, Azgp1, Bad, Bank1, Blnk, Btla, C3, Card11, Cbfb, Ccl1, Ccl5, Cd1d1, Cd1d2, Cd247, Cd28, Cd38, Cd40lg, Cd55, Cd79b, Cd80, Cebpg, Clcf1, Clec4a2, Clec4n, Csf1r, Csf1r, Cx3cr1, Cxcr4, Cxcr5, Ddx58, Dock2, Epas1, Fcer1g, Fcgr2b, Fkbp1b, G6pdx, Gapt, Gimap5, Gnas, H2-Aa, H2-Ab1, H2-DMa, H2-DMb1, H2-DMb2, H2-Ea, H2-Eb1, H2-T3, Hc, Hdac9, Hhex, Hoxb3, Hspa1b, Icam1, Igh-6, Ikbkg, Il16, Il1rap, Il2ra, Inhba, Irak1, Itgam, Kdr, Klhl6, Lat2, Lig4, Lst1, Ly6d, Lyn, Lyst, Mll5, Ms4a1, Mx1, Mx2, Myo1e, Myo1f, Nfam1, Nfkb1, Nlrx1, Oas1b, Orm2, Pbx1, Pdcd1lg2, Pecam1, Plcg1, Plcg2, Polm, Pou2f2, Prkca, Prkcd, Procr, Ptpn6, Ptprc, Rab27a, Rara, Rc3h1, Ripk2, Rogdi, Rsad2, S100a9, Selp, Sfpi1, Sh2b2, Sh2b3, Sh2d1a, Sh2d1b1, Slamf1, Slc11a1, Spib, Stap1, Stat6, Tac4, Tapbp, Tbk1, Tcfeb, Tgfbr2, Tinagl1, Tirap, Tlr1, Tlr2, Tlr4, Tlr7, Tnfaip8l2, Tnfrsf13b, Tnfrsf13c, Twsg1, Vnn1, Ywhaz, Fas, Fasl, Gzma, Gzmm
miR-10b	Ahsp, C3ar1, C4bp, Cd34, Cdk6, Cebpa, Crip2, Crkl, Ctla4, Cxcl9, Enpp3, Fyb, Gpam, H28, H2-Q1, H2-Q10,Hells, Hoxa9, Id2, Il12b, Il1r1, Il1rl1, Ilf2, Kit, Klf1, Masp2, Msh2, Pf4, Polr3h, Prg2, Prg3, Runx1, Serpinb9, Slc11a2, Sox6, Spna1, Tacc3, Tal1, Tap2, Tek, Tgtp1, Tlr3, Tnfsf9, Vpreb1, Cd28
miR-125a-3p	Ahsp, Alas2, Ank1, Bak1, Barx1,Bcl10, C3ar1, C4bp, C8a, Cd34, Cd47, Cdk6, Cebpa, Cplx2, Cr1l, Crip2, Crkl, Csf1, Ctla4, Ctse, Cxcl11, Cxcl13, Cxcl9, Daf2, Enpp2, Enpp3, Fech, Fyb, Gpam, H28, H2-Q1, H2-Q10, Hbb-b1, Hells, Heph, Hmgb1, Hoxa9, Icam1, Id2, Il12b, Il1r1, Il1rl1, Il2ra, Ilf2, Jarid2, Kcnj8, Kit, Klf1, Klf11, Klre1, Lrrc17, Masp1, Masp2, Mbl2, Mecom, Meis1, Msh2, Mx1, Nup85, Osm, Pf4, Pik3cd, Pml, Polr3h, Prg2, Prg3, Rag1, Runx1, Serpinb9, Serping1, Sfxn1, Sh2b2, Slc11a2, Sox6, Spna1, Tacc3, Tal1, Tap2, Tbx1, Tcf3, Tcfe3, Tek, Tgtp1, Tlr3, Tnfsf11, Tnfsf8, Tnfsf9, Traf3ip2, Txnrd2, Vegfa, Vpreb1, Cd28, Itgb6
miR-141	Ahsp, Alas2, Ank1, Bak1, Barx1, Bcl10, C3ar1, C4bp, C8a, Cd34, Cd47, Cdk6, Cebpa, Cplx2, Cr1l, Crip2, Crkl, Csf1, Ctla4, Ctse, Cxcl11, Cxcl13, Cxcl9, Daf2, Enpp2, Enpp3, Fech, Fyb, Gpam, H28, H2-Q1, H2-Q10, Hbb-b1, Hells, Heph, Hmgb1, Hoxa9, Icam1, Id2, Il12b, Il1r1, Il1rl1, Il2ra, Ilf2, Jarid2, Kit, Klf1, Klf11, Klre1, Lrrc17, Masp1, Masp2, Mbl2, Mecom, Meis1, Msh2, Mx1, Nup85, Osm, Pf4, Pik3cd, Pml, Polr3h, Prg2, Prg3, Rag1, Runx1, Serpinb9, Serping1, Sfxn1, Sh2b2, Slc11a2, Sox6, Spna1, Tacc3, Tal1, Tap2, Tbx1, Tcf3, Tcfe3, Tek, Tgtp1, Tlr3, Tnfsf11, Tnfsf8, Tnfsf9, Traf3ip2, Txnrd2, Vpreb1, Cd28
miR-149	Ahsp, Bak1, C3ar1, C4bp, C8a, Cd34, Cdk6, Cebp, Cplx2, Crip2, Crkl,Csf1, Ctla4, Ctse, Cxcl11, Cxcl13, Cxcl9, Enpp3, Fech, Fyb, Gpam, H28, H2-Q1, H2-Q10, Hbb-b1, Hells, Heph, Hoxa9, Icam1, Id2, Il12b, Il12b, Il1r1, Il1rl1, Il2ra, Ilf2, Kit, Klf1, Klf11, Lrrc17, Masp2, Meis1, Msh2, Mx1, Nup85, Osm, Pf4, Pik3cd, Pml,Polr3h, Prg2, Prg3, Rag1, Runx1, Serpinb9, Serping1, Sh2b2, Slc11a2, Sox6, Spna1, Tacc3, Tal1, Tap2, Tbx1, Tcf3, Tek, Tgtp1, Tlr3, Tnfsf8, Tnfsf9, Txnrd2, Vpreb1, Cd28
miR-1897-5p	Cdk6, Cxcl9, Elf4, Fech, Foxo3, Fyb, Fyb, Gpam, H28, H2-Q1, H2-Q10, Hoxa9, Il12b, Il12b, Il1r1, Il1rl1, Klf1, Masp2, Masp2, Polr3h, Slc11a2, Slc11a2, Smap1, Tap2, Tek, Tgtp1
MicroRNAs	Potencial targets
miR-29b*	Add2, Ahsp, Ank1, Bak1, C3ar1, C4bp, C8a, Cd34, Cdk6, Cebpa, Cplx2, Crip2, Crkl, Csf1, Ctla4, Ctse, Cxcl11, Cxcl13, Cxcl9, Elf4, Enpp3, Fech, Fech, Fech, Foxo3, Fyb, Gpam, H28, H2-Q1, H2-Q10, Hbb-b1, Hells, Heph, Hoxa9, Icam1, Id2, Il12b, Il1r1, Il1rl1, Il2ra, Il6, Ilf2, Kit, Klf1, Klf11, Lrrc17, Masp2,Meis1, Msh2, Mx1, Nup85, Osm, Pf4, Pik3cd, Pml, Polr3h, Prg2, Prg3, Rag1, Rorc, Runx1, Serpinb9, Serping1, Sh2b2, Slc11a2, Smap1, Sox6, Spna1, Spon2, Tacc3, Tal1, Tap2, Tbx1, Tcf3, Tek, Tgtp1, Thy1, Tlr3, Tnfsf8, Tnfsf9, Txnrd2, Vpreb1, Cd28
miR-302c	Ahsp, Cdk6, Crkl, Ctla4, Cxcl9, Fyb, Gpam, H28, H2-Q1, H2-Q10, Hoxa9, Il12b, Il1r1, Il1rl1, Kit, Klf1, Masp2, Msh2, Polr3h, Prg2, Prg3, Runx1, Serpinb9, Slc11a2, Spna1, Tal1, Tap2, Tek, Tgtp1, Cd28
miR-30b*	Add2, Ahsp, Alas2, Ank1, Bak1, Barx1, Bcl10, C3ar1, C4bp, C8a, Cd34, Cd47, Cdk6, Cebpa, Cplx2, Cr1l, Crip2, Crkl, Csf1, Ctla4, Ctse, Cxcl11, Cxcl13, Cxcl9, Daf2, Elf4, Enpp2, Enpp3, Fech, Foxo3, Fyb, Gpam, H28, H2-Q1, H2-Q10, Hbb-b1, Hells, Heph, Hoxa9, Icam1, Id2, Il12b, Il1r1, Il1rl1, Il2ra, Il6, Ilf2, Jarid2, Kit, Klf1, Klf11, Klre1, Lrrc17, Masp1, Masp2, Mbl2, Mecom, Meis1, Msh2, Mx1, Nup85, Osm, Pf4, Pik3cd, Pml, Polr3h, Prg2, Prg3, Rag1, Rorc, Runx1, Serpinb9, Serping1, Sfxn1, Sh2b2, Slc11a2, Smap1, Sox6, Spna1, Tacc3, Tal1, Tap2, Tbx1, Tcf3, Tcfe3, Tek, Tgtp1, Thy1, Tlr3, Tnfsf8, Tnfsf9, Traf3ip2, Txnrd2, Vpreb1, Bcl2, Cd28
miR-690	Add2, Ahctf1, Ahsp, Alas2, Ank1, Bak1, Bcl10, C3ar1, C4bp, C8a, Cd34, Cdk6, Cebpa, Cplx2, Crip2, Crkl, Csf1, Ctla4, Ctse, Cxcl11, Cxcl13, Cxcl9, Daf2, Dyrk3, Elf4, Enpp3, Fech, Foxo3, Fyb, Gpam, H28, H2-Q1, H2-Q10, Hbb-b1, Hells, Heph, Hmgb3, Hoxa9, Icam1, Id2, Il12b, Il12b, Il18r1, Il1r1, Il1rl1, Il2ra, Il6, Ilf2, Kit, Klf1, Klf11, Lrrc17, Masp2, Meis1, Msh2, Mx1, Ncaph2, Nup85, Osm, Pf4, Pik3cd, Pml, Polr3h, Prdx2, Prg2, Prg3, Rag1, Rorc, Runx1, Serpinb9, Serping1, Sh2b2, Slc11a2, Slc11a2, Smap1, Sox6, Spna1, Spon2, Tacc3, Tal1, Tap2, Tbx1, Tcf3, Tcfe3, Tek, Tgtp1, Thy1, Tlr3, Tnfsf8, Tnfsf9, Traf3ip2, Txnrd2, Vpreb1, Bcl2, Cd28
miR-691	Ahsp, C3ar1, C4bp, Cd34, Cdk6, Cebpa, Crip2, Crkl,Ctla4, Ctse, Cxcl11, Cxcl9, Enpp3, Fyb, Gpam, H28, H2-Q1, H2-Q10, Hells, Hoxa9, Icam1, Id2, Il12b, Il1r1, Il1rl1, Il2ra, Ilf2, Kit, Klf1, Masp2, Msh2, Osm, Pf4, Polr3h, Prg2, Prg3, Runx1, Serpinb9, Slc11a2, Sox6, Spna1, Tacc3, Tal1, Tap2, Tek, Tgtp1, Tlr3, Tnfsf9, Vpreb1, Cd28
miR-712	Ahsp, C3ar1, C4bp, Cd34, Cdk6, Cebpa, Crip2, Crkl, Ctla4, Ctse, Cxcl11, Cxcl9, Enpp3, Fyb, Gpam, H28, H2-Q1, H2-Q10, Hells, Hoxa9, Icam1, Id2, Il12b, Il1r1, Il1rl1, Il2ra, Ilf2, Kit, Klf1, Masp2, Msh2, Osm, Pf4, Pml, Polr3h, Prg2, Prg3, Runx1, Serpinb9, Slc11a2, Sox6, Spna1, Tacc3, Tal1, Tap2, Tek, Tgtp1, Tlr3, Tnfsf9, Vpreb1, Cd28

During the initial stage of T cell maturation in the thymus and their migration to the periphery, we found few potential targets related to immune functions when compared to activated T cells during immunization (Table 1). In general, the up-regulated miRNAs found in the DBA-2/J strain regulated higher numbers of targets (884 mRNA targets) than those found in the DBA-1/J strain (521 mRNA targets).

**Figure 5:**
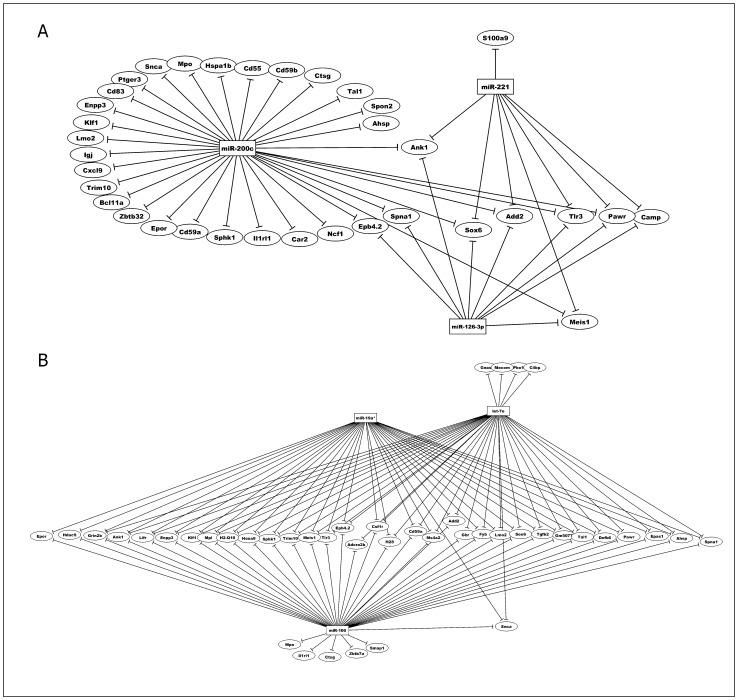
Post-transcriptional miRNA-mRNA interaction networks based on microarray expression data from thymocytes. The networks were obtained using the GenMir++ algorithm. This figure shows the participation of A) three miRNAs up-regulated in thymocytes of DBA-1/J strain and B) three miRNAs in up-regulated in thymocytes of DBA-2/J. These miRNAs interact with mRNAs related to immune system.

Interestingly, in thymocytes of both mouse strains, two different sets of miRNAs regulated a common set of targets, the Sox6, Tlr3 and Add2 mRNAs (Figure 5).

**Figure 6:**
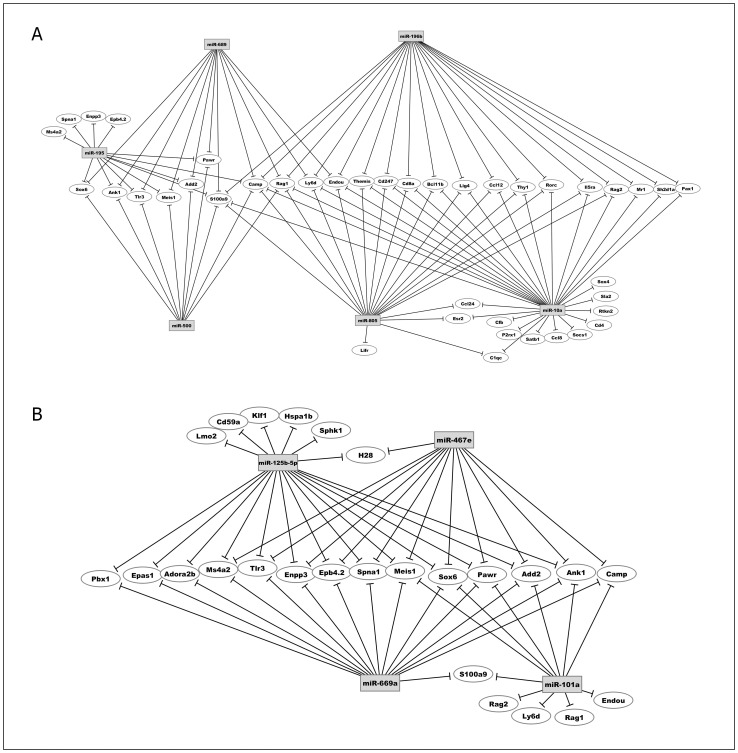
Post-transcriptional miRNA-mRNA interaction networks based on microarray expression data from peripheral CD3^+^ T cells. The networks were obtained using the GenMir++ algorithm. This figure shows the participation of A) six miRNAs up-regulated in CD3+ T cells of DBA-1/J strain and B) four miRNAs up-regulated in CD3+ T cells of DBA-2/J strain. These miRNAs interact with mRNAs related to immune system.

As expected, the peripheral mature T lymphocytes from these strains showed regulation of the Rag1, Rag2, and Themis mRNAs, which are associated with T cell differentiation and maturation (Figure 6).

**Figure 7:**
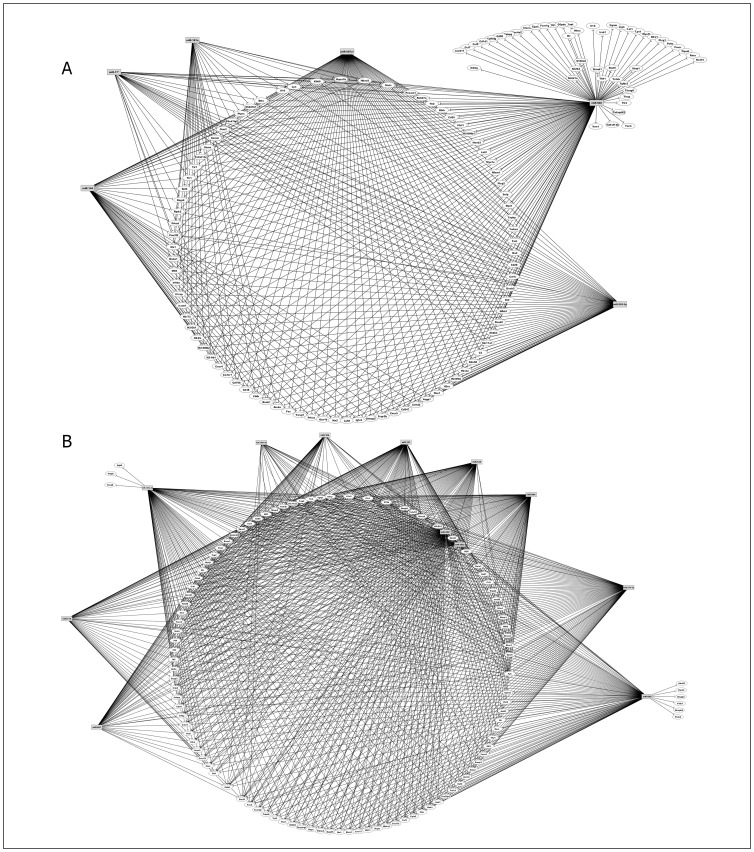
Reduced percentage of CD8^+^ T cells during collagen induced arthritis (CIA) in the DBA-1/J susceptible strain. The percentage of splenic or lymph nodes T cells of DBA-1/J and DBA-2/J mice expressing CD8a was evaluated by flow cytometry. Data from three independent experiments are shown. The results are presented as standard error of the mean (SEM). The differences were evaluated by Student’s t-test. **P<0.01 was considered statistically significant in DBA-2/J mice strain when compared to DBA-1/J.

Figure 6a shows that miRNAs miR-196b, miR-805 and miR-10a regulated CD8a mRNA in the peripheral T cells of the DBA-1/J strain. Flow cytometry analysis shows that the percentage of peripheral CD8^+^ T cells is lower in DBA-1/J mice than in DBA-2/J mice (Figure 7), which suggest the participation of these miRNAs as negative regulatory controllers.

**Figure 8:**
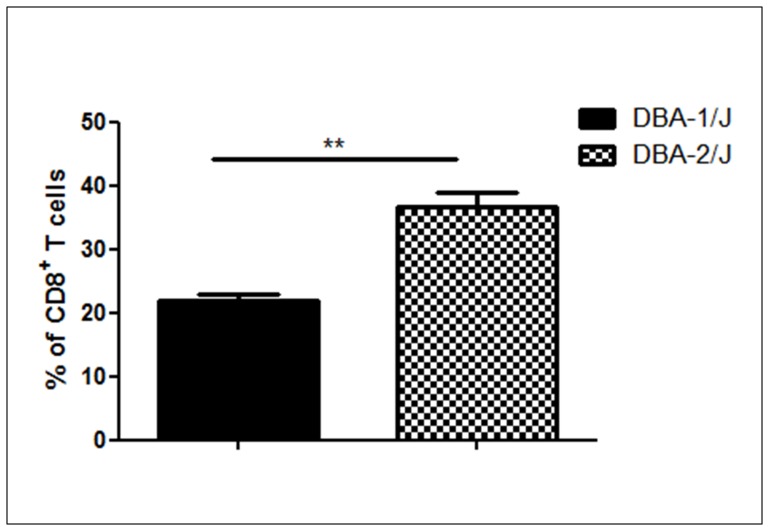
Post-transcriptional miRNA-mRNA interaction networks based on microarray expression data from peripheral CD3^+^ T cells during collagen induced arthritis (CIA). The networks were obtained using the GenMir++ algorithm. This figure shows the participation of A) six miRNAs up-regulated in peripheral CD3+ T cells of DBA-1/J mice and B) eleven miRNAs up-regulated in peripheral CD3+ T cells of DBA-2/J mice. These miRNAs interact with mRNAs related to immune system. The control mice were immunized just with complete Freund’s adjuvant without any collagen.

During CIA development in DBA-1/J mice, the number of mRNA targets increased, which included important mRNAs such as Fas and FasL that encode apoptosis proteins, IL-10, Stat6, and granzymes Gzmm and Gzma (Figure 8a).

**Figure 9:**
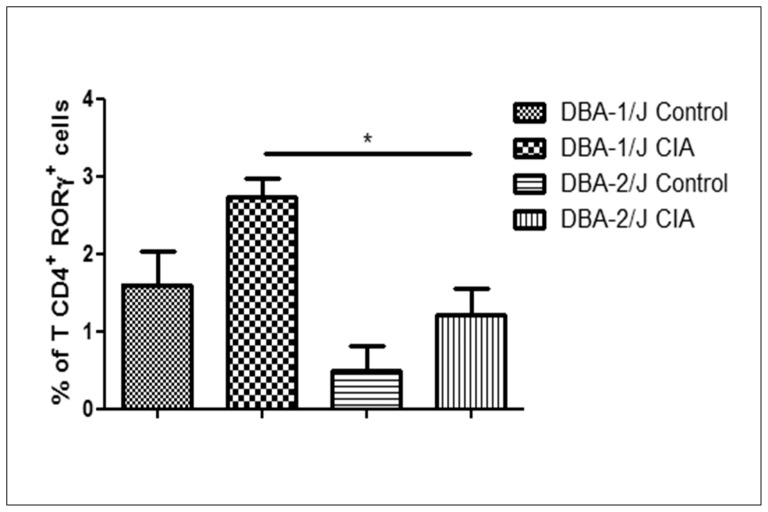
Reduced percentage of Rorγt^+^ T cells during collagen immunization of DBA-2/J strain. The percentage of splenic or lymph node expressing RORγt T cells of DBA-1/J and DBA-2/J mice during immunization with type II collagen was evaluated by flow cytometry. The control mice were immunized just with complete Freund’s adjuvant without any collagen. Data from three independent experiments are shown. The results are presented as means standard error of mean (SEM). The differences were evaluated by a one-way ANOVA followed by Bonferroni’s test. *P<0.05 was considered statistically significant in DBA-2/J mice strain when compared to DBA-1/J after CIA induction.

However, during collagen immunization of DBA-2/J mice, the mRNA targets Rorc, IL-6, Foxo3 and Bcl2 were found (Figure 8b). Because Th17 cells are strongly correlated with arthritis development, we highlighted the Rorc mRNA, which encodes the transcription factor Rorγt implicated in the differentiation of these cells. Rorc mRNA was regulated by miR-30b*, miR-690 and miR-29b* (Table 1). Our flow cytometry analysis showed that the percentage of CD4^+^ Rorγt^+^ T cells was lower in the DBA-2/J strain (Figure 9), and again the participation of these miRNAs as negative regulatory controllers is suggested.

**Figure 10:**
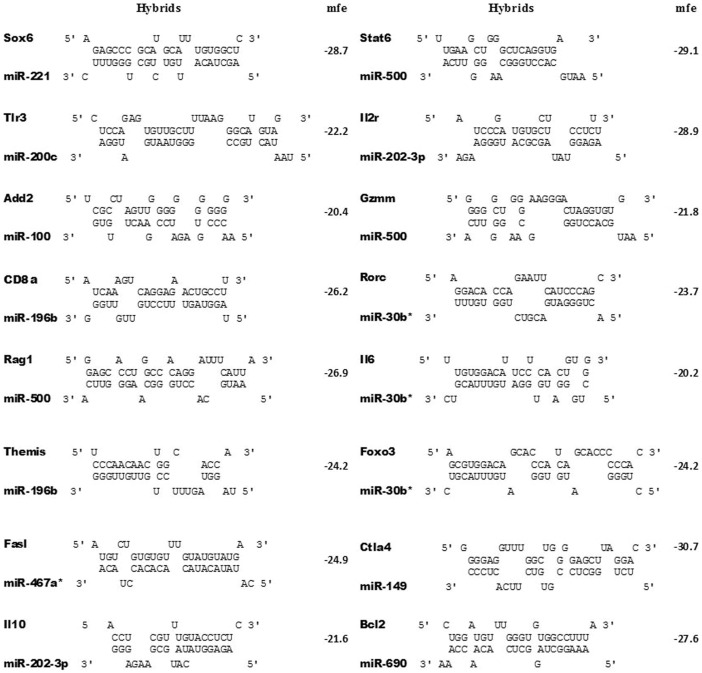
Validation of miRNA-mRNA interactions using RNAhybrid software. The miRNA:mRNA hybrid structures were calculated for selected examples of miRNA-mRNA interactions from GenMir++ predictions. Sixteen miRNA:mRNA hybrid structures are presented. The miRNA binding site of the target mRNA is shown at the top, the complementary miRNA is shown at the bottom strand, and the calculated minimum free energy (MFE) values are given to the right (kcal/mol). The gene names as well as the designation of the miRNA (miRBase) are shown to the left.

As the most of the miRNA-mRNA interactions found in this study are new, we validated some of such using the RNAhybrid bioinformatic tool, which shows the most favorable hybridization between a given miRNA and its predicted mRNA target by calculating the minimum free energy (MFE). The miRNA-mRNA molecular interactions and respective MFEs are shown on Figure 10.

**Figure 11:**
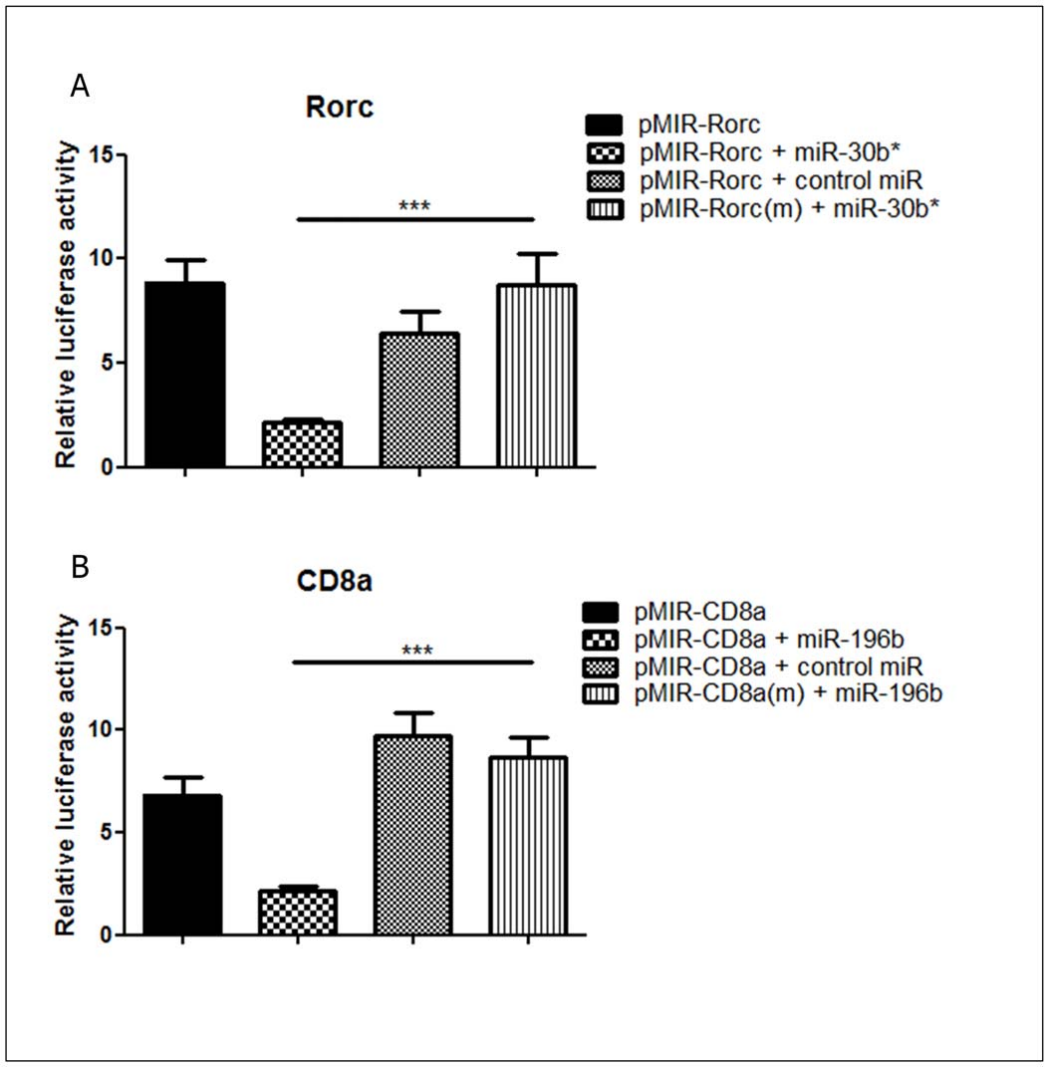
Luciferase reporter gene assay. pMIR-Rorc and pMIR-Rorc(m) 3′UTR luciferase plasmid were co-transfected with control or miR-30b* mimic (A), and pMIR-CD8a and pMIR-CD8a(m) with control or miR-196b (B) into HEK293 cells. Luciferase activity was evaluated at 24 h post transfections. Data are presented as means standard error of mean (SEM). The differences were evaluated by a one-way ANOVA followed by Bonferroni’s test. ***P<0.001 was considered statistically significant when the wild-type pMIR was compared to the mutant mismatch sequence in the presence of the respective miRNA mimic.

Moreover, the luciferase reporter assay was useful to confirm the occurrence of miRNA-mRNA interactions within the cellular milieu focusing on Rorc and CD8a mRNAs targets. We confirmed that miR-30b* interacts with Rorc mRNA and miR-196b interacts with CD8a mRNA by hybridization with their respective 3′UTR sequences containing the predicted binding sites for these miRNAs. Introduction of point mutations into the 3′UTR targeted sequences significantly abolished miRNA hybridization (Figure 11).

## Discussion

Rheumatoid arthritis (RA) is an autoimmune disease that is characterized by an uncontrolled influx of inflammatory T cells to the joints, eventually leading to tissue damage. Pro- and anti-inflammatory cytokines and chemokines and/or their receptors, which are key mediators of the effector function of T cells, appear to be centrally involved in the pathogenesis of RA [1].

The role that T cells play in inflammation and joint damage has been elucidated through the usage of animal models, mainly mice [40]. The DBA-1/J and DBA-2/J strains of mouse, which are respectively susceptible and resistant to CIA [41], represent in these days a unique model-system for studying the mechanisms involved in arthritis susceptibility.

Although the main effector cells and proteins involved in human RA and experimental murine CIA have already been identified, efforts to better understand its molecular and genetic control are just beginning. In this regard, our group recently demonstrated that thymic stroma of the DBA-1/J and DBA-2/J strains differ in their transcriptional expression of joints, cartilage and bone peripheral tissue antigens (PTAs). Interestingly, in the DBA-1/J CIA-susceptible strain, the MHC-H2 gene represents a key regulator of downstream genes that code joints and bone PTAs [10]. We show that MHC-H2 at least partly control the expression of autoantigens in the thymus, a phenomenon known as promiscuous gene expression that is important for the negative selection of autoreactive thymocytes [42]–[44].

In the present study, we asked whether thymocytes, T cells in different phases of development and activation, naïve and activated peripheral CD3^+^ T lymphocytes in the context of CIA, also featured differences in their transcriptional expression of miRNAs and mRNAs.

Considering the role played by miRNAs in the post-transcriptional control of gene expression in immune cells [45], we also asked whether these molecules could differentially interact with mRNA targets. To test this, we used the microarray method and bioinformatics programs to trace gene expression signatures and miRNA-mRNA interactions (Figure S1).

Initially we conducted genome-wide transcriptional profiling of miRNAs and mRNAs, as depicted in Figures 1–4.

When thymocytes where compared to naïve T cells, we noticed that some miRNAs, including miR-30e, miR-363 and miR-130b, did not show altered expression. We also found T cell developmental stage-specific miRNAs with no difference between DBA-1/J and DBA-2/J mouse strains. This suggests that these miRNAs were related to common processes between the two strains. As our aim was to identify the miRNAs associated with susceptibility or resistance to CIA, then we focused our analysis on cell-type and strain-specific miRNAs.

We show the clustering of peripheral CD3^+^ T lymphocyte miRNA profiles from the DBA-1/J and DBA-2/J strains after collagen immunization. Interestingly, most of the miRNAs differ in their expression profile between the mouse strains. The miR-223 among some other represents exception of miRNAs that were induced in both strains.

The mRNA expression profile of thymocytes and peripheral CD3^+^ T lymphocytes from the DBA-1/J and DBA-2/J strains was also compared. Using Gene Ontology (GO), we identified those mRNAs that are related to the immune system due to their importance to arthritis. GO allowed identifying 1,813 immune system-related mRNAs in thymocytes and naïve T cells and 486 in CD3^+^ T cells during collagen immunization of DBA-1/J and DBA-2/J mouse strains (Figures 3–5).

These results were important in demonstrating that the transcriptional programming of T cells is somewhat different between the mice strains studied, answering the first question of this study.

The hierarchical clustering of microarray data is very useful in grouping biological samples and represents the gold standard method. However, this does not address the possibility that the differentially expressed RNAs are somehow interacting with each other. As our second question addressed on the miRNA-mRNA interactions, we had to use a dedicated method for estimating this. Therefore, we choose the GenMir^++^ program [38], which establishes network interactions based on Bayesian statistics of opposite expression levels of miRNAs and mRNAs from actual microarray data. The set of miRNA and mRNA microarray data was then inputted to this program, which was used to identify potential targets through reconstruction of miRNA-mRNA interaction networks.

In general, miRNA-mRNA target pairs are penalized if both the miRNA and mRNA are highly expressed in the same sample and are rewarded if a given miRNA is highly expressed in a sample that a given mRNA has low expression in, especially if few of its other predicted miRNA regulators are highly expressed therein.

Each data set of differentially expressed RNAs (miRNAs and mRNAs) from the same sample was analyzed together to reconstruct interaction networks. The reconstructed networks show that one given miRNA can interact with several mRNA targets and that one mRNA can interact with several miRNAs at once. The interactions shown in these figures are clearly identified in Table 1.

During T cell maturation in the thymus of DBA-1/J and DBA-2/J mice, potential mRNA targets related to the immune system were found, including Sox6 Tlr3, and Add2. Despite the differential miRNA profiles of thymocytes from the two mouse strains studied, they do have common mRNA targets in addition to exclusive targets. Because of this, we next asked if transcriptional deregulation in the periphery could be associated with CIA.

Although the number of mRNA targets in naïve T cells was low, interesting differences were observed. For example, the Rag1, Rag2 and Themis mRNAs, which are involved in the differentiation and maturation of thymocytes, were regulated in both strains (Figure 6). Themis is expressed in a tightly regulated manner during T cell development in late double-negative (DN) and especially in double-positive (DP) thymocytes [46]. It is down-regulated after positive selection and is expressed at low levels in mature T cells. As expected, we found that genes involved with early T cell differentiation processes, Rag1, Rag2 and Themis, were regulated by miRNAs and consequently down-regulated in the periphery.

Another interesting mRNA target regulated in the DBA-1/J strain is the Cluster of differentiation 8 (CD8). CD8^+^ T cells seem to be important in the effector phase of arthritis disease but are dispensable for its initiation [47]. However, the importance of the CD8^+^ T cells in arthritis and their role in induction or protection are still controversial. Interestingly, we found that the DBA-1/J strain showed lower numbers of CD8^+^ T cells in the periphery compared with the DBA-2/J strain (Figure 7).

Figure 8 shows the miRNA-mRNA interactions in the DBA-1/J and DBA-2/J strains during CIA development. Among the mRNAs differentially expressed and downregulated in peripheral CD3^+^ T cells during collagen immunization and CIA development in DBA-1/J mice, we found genes that positively regulate inflammation, including Fas and FasL involved with apoptosis and IL-10 involved in suppressing Treg function.

Fas and FasL are part of the activation-induced cell death (AICD) pathway involved in the removal of activated T cells in vivo [48], [49]. IL-10 production by Tregs is one of the mechanisms involved in inhibiting the production of inflammatory mediators and lymphocyte proliferation [50], [51]. Our results suggest that the strong regulation of these genes in the CIA-susceptible DBA-1/J strain might contribute to the exacerbated T cell response observed in CIA.

Among the targets that are down-regulated during collagen immunization of the DBA-2/J strain, we found Rorc, which is involved in Th17 differentiation, and Foxo3, which is involved in cell cycle and survival, both of which are directly related to arthritis pathogenesis. In fact, Foxo3 has already been associated with RA. Foxo3 mRNA is increased in patients, and its overexpression may lead to the prolonged T cell survival in RA, thereby contributing to chronic inflammation [52].

Rorc mRNA encodes the transcription factor Rorγt, which is involved in the differentiation of CD4^+^ T cells to Th17 pro-inflammatory cells. The role of IL-17 in the pathogenesis of arthritis is well known and was first confirmed in the CIA model [53]. In agreement with the FACS results of CD8^+^ naïve T cells, we also found that the CIA-resistant DBA-2/J strain has diminished numbers of Rorγt^+^T cells in the periphery (Figure 9).

An intriguing result from this study was the finding that Rorc mRNA was regulated by miR-500 in naïve T cells in the DBA-1/J strain. However, during collagen immunization and CIA development in this strain, none miRNA regulation upon this mRNA was observed. In contrast, in DBA-2/J mice, Rorc mRNA expression was regulated by miR-29b*, miR-30b* and miR-690.

To the best of our knowledge, only miR-181a and miR-221, from the interaction networks showed in this study, have been previously identified as playing a role in T lymphocytes [26], [54]. Moreover, we found new miRNAs that were up-regulated during the development and activation of T lymphocytes in the context of susceptibility or resistance to CIA.

Finally, we highlight the validation in this study of the miRNA-mRNA interactions that were not previously predicted in data bases such as TargetScan: miR-30b*-Rorc and miR-196b-CD8a. These interactions were initially validated by minimal free energy calculations of the respective RNA-RNA hybridizations. We used the RNAhybrid program for this purpose, which allowed us to know the specific miRNA-mRNA annealing sequence (Figure 10).

The portion of the mRNA 3′UTR sequences (annealing sequence) identified through RNAhybrid program was then synthesized, cloned in the pmirGlo vector, which was designed to assay the miRNA-mRNA interaction within the cell milieu (luciferase reporter assay). This assay allowed confirming the in vivo occurrence of the two new miRNA-mRNA interactions studied, which represent important pieces in the study of susceptibility/resistance to CIA. For each miRNA-mRNA interaction, the minimum free energy (MFE) was calculated based on the respective thermodynamic state (Figure 10). We used an MFE threshold of -20 kcal/mole based on the interaction between miRNAs with lengths of 22 nucleotides (nt) and a target with a length of 1,000 nt [33].

In this study, we identified a set of new miRNAs that regulate mRNA targets in different stages of T cell differentiation and activation in the context of murine CIA. These miRNAs differed in their expression profiling between DBA-1/J and DBA-2/J mice, which are susceptible and resistant to CIA, respectively. The modulation of miRNA expression allowed for the identification of miRNA-mRNA interactions, which were visualized through networks reconstructed based on actual microarray transcriptional profile and then validated through luciferase assay (Figure 11).

Important mRNA targets related to T cell differentiation and activation were predicted, including those related to inflammatory processes (Rorc), which were strongly regulated in the CIA-resistant DBA-2/J strain. The mRNAs involved in the control of an aggressive immune response, such as Fas and FasL, were found as miRNA targets in the CIA susceptible DBA-1/J strain.

The meaningful of these findings is reinforced since the mRNAs identified in this study encode proteins related to the pathogenesis of arthritis. Moreover, our results suggest a deregulation of miRNAs that could be associated with the control of molecules involved in inflammation development in the susceptible DBA-1/J strain following CIA induction.

These results contribute to a better understanding of the transcriptional and post-transcriptional modulation that might be contributing to the susceptibility/resistance to CIA in mice.

## Supporting Information

Figure S1 **Experimental design.** Workflow for discriminating between the biological samples used (animal groups and cell types), total RNA extraction, hybridizations, microarray data analysis and miRNA-mRNA validation.
(TIF)
