# Correction: T Cell Post-Transcriptional miRNA-mRNA Interaction Networks Identify Targets Associated with Susceptibility/Resistance to Collagen-induced Arthritis

**DOI:** 10.1371/annotation/d5b49a6b-b176-4e96-9f4f-113838f0889d

**Published:** 2013-06-14

**Authors:** Paula B. Donate, Thais A. Fornari, Claudia Macedo, Thiago M. Cunha, Daniele C. B. Nascimento, Elza T. Sakamoto-Hojo, Eduardo A. Donadi, Fernando Q. Cunha, Geraldo A. Passos

The legends for Figures 7 and 8 were swapped. The legend that appears with Figure 7 actually corresponds to Figure 8, and the legend that appears with Figure 8 actually corresponds to Figure 7. 

