# Supplementary figures and images for: T Cell Post-Transcriptional miRNA-mRNA Interaction Networks Identify Targets Associated with Susceptibility/Resistance to Collagen-induced Arthritis
Source: PLoS One. 2013 Jan 24;8(1):e54803. doi: 10.1371/journal.pone.0054803 (PMC3554629; doi:10.1371/journal.pone.0054803)

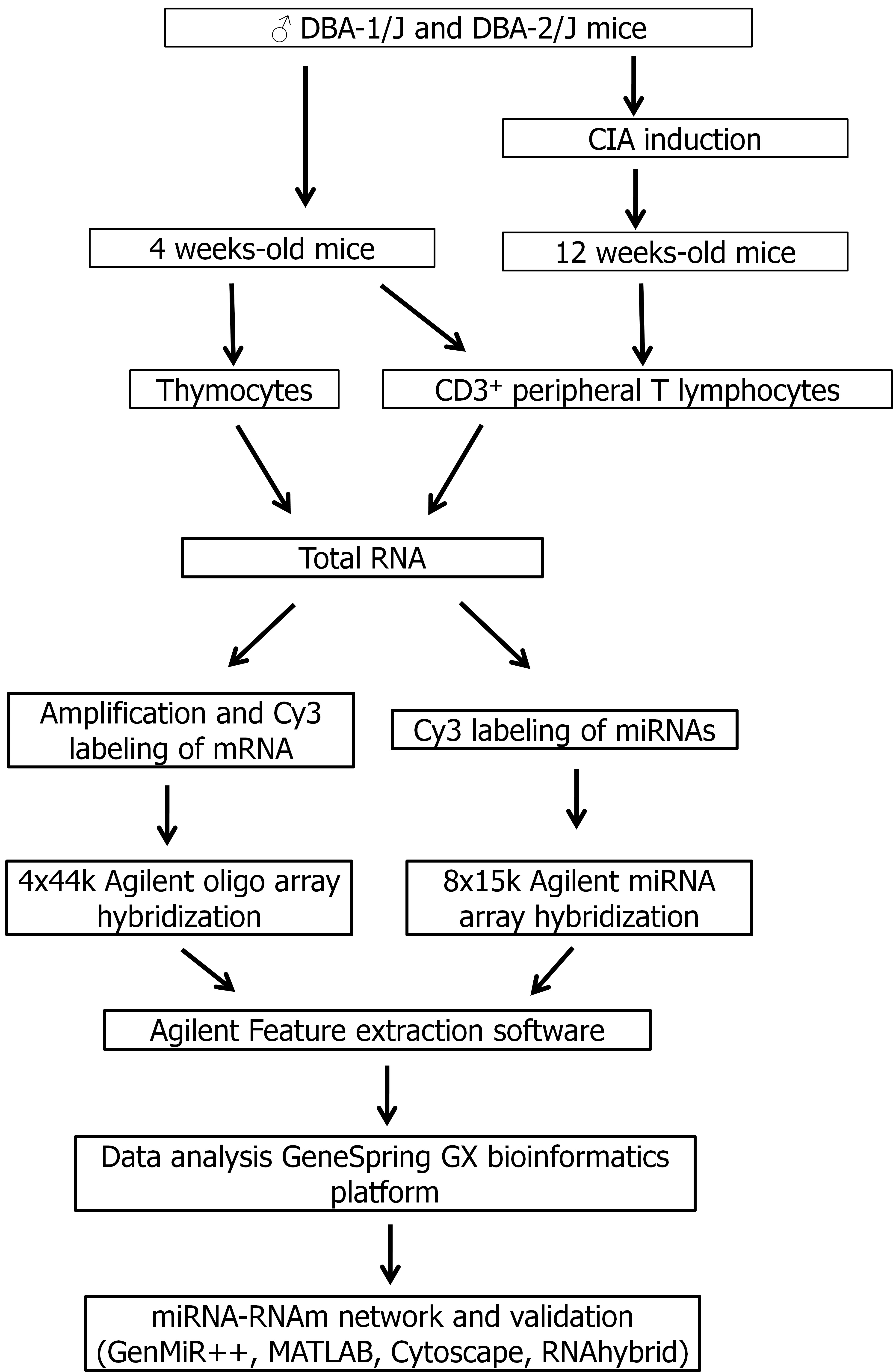

Supplement: Figure S1 — Experimental design. Workflow for discriminating between the biological samples used (animal groups and cell types), total RNA extraction, hybridizations, microarray data analysis and miRNA-mRNA validation. (TIF) [file pone.0054803.s001.tif]
